# Centralized registry for COVID-19 research recruitment: Design, development, implementation, and preliminary results

**DOI:** 10.1017/cts.2021.819

**Published:** 2021-07-14

**Authors:** Anna Peeler, Hailey Miller, Oluwabunmi Ogungbe, Cassia Lewis Land, Liz Martinez, Monica Guerrero Vazquez, Scott Carey, Sumati Murli, Megan Singleton, Cyd Lacanienta, Kelly Gleason, Daniel Ford, Cheryl R. Himmelfarb

**Affiliations:** 1 Johns Hopkins University School of Nursing, Baltimore, MD, USA; 2 Johns Hopkins University Institute for Clinical and Translational Research, Baltimore, MD, USA; 3 Duke University School of Nursing, Durham, NC, USA; 4 Johns Hopkins School of Medicine, Baltimore, MD, USA; 5 Center for Salud/Health and Opportunity for Latinos, Baltimore, MD, USA

**Keywords:** COVID-19, recruitment, registry, clinical trials, community engagement

## Abstract

**Background:**

The Coronavirus Disease 2019 (COVID-19) pandemic has had substantial global morbidity and mortality. Clinical research related to prevention, diagnosis, and treatment of COVID-19 is a top priority. Effective and efficient recruitment is challenging even without added constraints of a global pandemic. Recruitment registries offer a potential solution to slow or difficult recruitment.

**Objectives:**

The purpose of this paper is to describe the design and implementation of a digital research recruitment registry to optimize awareness and participant enrollment for COVID-19-related research in Baltimore and to report preliminary results.

**Methods:**

Planning began in March 2020, and the registry launched in July 2020. The primary recruitment mechanisms include electronic medical record data, postcards distributed at testing sites, and digital advertising campaigns. Following consent in a Research Electronic Data Capture survey, participants answer questions related to COVID-19 exposure, testing, and willingness to participate in research. Branching logic presents participants with studies they might be eligible for.

**Results:**

As of March 24, 2021, 9010 participants have enrolled, and 64.2% are female, 80.6% are White, 9.4% are Black or African American, and 6% are Hispanic or Latino. Phone outreach has had the highest response rate (13.1%), followed by email (11.9%), text (11.4%), and patient portal message (9.4%). Eleven study teams have utilized the registry, and 4596 matches have been made between study teams and interested volunteers.

**Conclusion:**

Effective and efficient recruitment strategies are more important now than ever due to the time-limited nature of COVID-19 research. Pilot efforts have been successful in connecting interested participants with recruiting study teams.

## Introduction

Since the end of 2019, the novel coronavirus has spread around the world, and no country has seen case numbers as consistently high as the USA.^1^ As of March 24, 2021, nearly 30 million Americans have contracted Coronavirus Disease 2019 (COVID-19), the disease caused by the novel coronavirus, and over 500,000 have died as a result of the disease.^2^ According to Centers for Disease Control and Prevention data, Hispanic individuals and non-Hispanic Black individuals are three times more likely to contract the coronavirus and are 4.7 times more likely to be hospitalized as a result of the disease than non-Hispanic Whites.^3^ In an effort to contain the disease and reduce the deleterious impact of the virus, clinical research urgently aimed at developing effective therapies and strategies to prevent, diagnose, and treat COVID-19 has become critical.

Effective and efficient patient recruitment is essential when conducting clinical research.^4–9^ Recruitment often represents a significant portion of a study’s overall budget and timeline, and failure to recruit an adequate sample can lead to expensive delays and underpowered results.^7,10^ Nearly one-fifth of clinical trials have been forced to terminate early due to inadequate timely recruitment, and nearly 90% of clinical trials fail to reach their recruitment goals in their expected timeline.^4^ Even in the best of scenarios, effective and efficient participant recruitment can be a challenge.^10^ During a pandemic when hundreds of thousands of lives are potentially at stake and millions more are on hold until the virus is under control, delays in research can bear even greater consequences.^6^

Recruitment registries offer a potential solution to the problem of slow or difficult recruitment. In the past, recruitment registries have been successful in recruitment of participants and connecting them with recruiting studies.^11^ An Alzheimer’s registry launched in 2015 has successfully enrolled over 75,000 research participants with a wide variety of risk factors that meet enrollment criteria of various research studies across multiple study teams.^12^ A team at Vanderbilt created a general recruitment registry, ResearchMatch, which has amassed over 150,000 participants since 2009 and has aided 834 study teams in achieving their recruitment goals.^13^ Recruitment registries have been particularly successful in connecting study teams to participants in niche or traditionally hard to reach patient populations like those with prostate cancer or older adults.^14,15^

COVID-19 research is currently a top global priority, and research teams are racing to try to reach potential participants and complete these studies efficiently. As such, we envisioned the development of a COVID-19 registry to serve as an essential tool to aid study teams in achieving their recruitment goals. Moreover, it would inform patients and community members about studies for which they might be eligible, while also preventing potential participants from being overwhelmed by outreach from multiple study teams.

The purpose of this paper is to describe the development and implementation of the Hopkins Opportunities for Participant Engagement (HOPE) Registry, a comprehensive, digital COVID-19 registry, and to report the preliminary recruitment results.

## Methods

### Registry Development

The development of the HOPE Registry began in early March when researchers from the Johns Hopkins Institute for Clinical and Translational Research (ICTR) recognized a need to expedite and regulate participant recruitment for multiple COVID-19-related studies competing for a limited number of eligible participants. At the time, there were no other recruitment registries at our institution, so work began to devise a system that could connect actively recruiting study teams with eligible and willing participants. While existing recruitment registries, like ResearchMatch and Pitt+Me, provided excellent examples of how registries should function, we chose to create a new system that fit the needs of the researchers at our institution and the Baltimore community.^13,16^

The development of the Registry infrastructure and the survey questions was led by the HOPE Advisory Council, which included the Vice Deans for Clinical Research from the School of Nursing and Medicine, the Associate Dean of Human Research Protections, faculty members from the Division of Infectious Diseases, senior informatics managers, research participant advocates, and expert staff serving in various research team roles. These faculty and staff were chosen to ensure that the registry met the needs of researchers, as well as patient populations across the academic health system. In addition to these individuals, key stakeholders from the Johns Hopkins ICTR Recruitment Innovation Unit and the Core for Clinical Research Data Acquisition (CCDA), the Community Research Advisory Council (C-RAC), the Center for Salud/Health and Opportunity for Latinos (Centro SOL), and faculty and staff from the Schools of Medicine, Nursing, and Public Health were consulted throughout the development of the Registry. Patient and community stakeholders provided input in all phases of registry development, implementation, and evaluation.

The project was approved by the Johns Hopkins Medicine Institutional Review Board in June 2020. The primary goal of the registry was to connect interested volunteers with outpatient COVID-19 research studies for which they might be eligible. Secondary goals of the registry were to: (1) increase awareness within and around Baltimore for current COVID-19-related research at our institution; (2) prevent potential participants from being contacted multiple times by various study teams; (3) allow participants autonomy in choosing a study that fits their needs; and (4) expand digital recruitment strategies at a time when in-person recruitment was impractical for outpatient studies.

The consent language, questions that comprise the registry survey, outreach messages, and advertisements were developed and refined with the help of the C-RAC, an advisory council made up of patients and community members whose goal is to improve community engagement throughout the research process. Centro SOL contributed to the co-development of a Spanish language option within the registry and recruitment materials.

### Study Team Enrollment

Information about the HOPE Registry is featured on the ICTR website to promote awareness among study teams. Additionally, members of the HOPE Registry team presented information about how to utilize the registry for recruitment purposes at various research-related events at our institution, including COVID-19 forums, grand rounds, and webinars. Word of mouth between study coordinators and teams has also been an effective strategy to spread the word about the HOPE Registry.

Study teams interested in recruiting participants through the HOPE Registry complete a brief registration form. Here, they are asked to indicate the eligibility criteria for their study, including the demographic information of interest and COVID-19-specific eligibility criteria such as exposure status, present symptoms, COVID-19 tests, and COVID-19 positivity status. The registry serves participants who speak Spanish, and study teams are required to indicate whether they could provide their study information in Spanish and provide continued language support and translation services to Spanish-speaking participants recruited through the registry. Study teams are also required to provide information on whether compensation, transportation cost reimbursement, or other incentives were provided in their study. The information requested of study teams is used to match participants and not intended to replace the extensive screening procedures for most studies, prior to enrollment; study teams are encouraged to indicate the most important eligibility criteria for the purpose of matching in the registry.

The HOPE Registry team reviews the study registration form to determine the appropriateness for the respective study for registry usage. Studies whose recruitment needs cannot be met through the registry, like studies seeking pediatric participants for example, are redirected to alternate pathways for recruitment facilitated by the Johns Hopkins ICTR. If approved, documentation of approval is issued to the study team for submission to the IRB either in the original application or as a change in research. The HOPE Registry team uses each study’s eligibility criteria to create appropriate branching logic in Research Electronic Data Capture (REDCap).^17,18^ Upon IRB approval, the branching logic is put into production in REDCap. Then, when a registry participant’s survey responses match the requirements of the branching logic, the participant is presented with the study description that was submitted in the registration form. The study description is also used to send Sway advertisements, described below.

## Participant Recruitment

To enroll, registry participants must be 18 years or older, willing and able to give informed consent, and not currently hospitalized. Participants are required to live within the USA.

### Recruitment Methods

In an effort to reach a large number of community members, the HOPE Registry employed a multi-pronged recruitment approach. First, adults who had been tested for COVID-19 at Johns Hopkins Health Systems testing sites were identified via a computable phenotype in the electronic medical record (EMR). Individuals with an active patient portal account were sent a patient portal message, regardless of testing result. Individuals that tested positive for COVID-19 in the last 24 hours were also contacted through email and/or text message, depending on the contact information available in the EMR. For individuals who did not have a mobile number in the EMR, trained recruitment navigators called their home phone. Beginning in September of 2020, a Spanish-speaking navigator contacted individuals whose preferred language was listed as Spanish in the EMR. Potential participants were contacted by no more than two methods. While our recruitment efforts primarily target those who have been tested for COVID-19, testing is not a requirement for joining the HOPE Registry. Anyone who meets the aforementioned inclusion criteria who would like to join the registry is permitted to do so.

To reach individuals outside of the Johns Hopkins Health System, physical and online advertisements were employed. Specifically, postcards were distributed at COVID-19 testing sites throughout the Baltimore area and online ads were circulated on Google, Facebook, and Instagram.

Each recruitment message included language about: the purpose of the HOPE Registry, invitation to consider participation in research related to the prevention, diagnosis, or treatment of COVID-19, and an assertion that participation is voluntary and will not affect the medical care they receive. Additionally, each method included a unique URL for the HOPE Registry survey that provided detailed information about how each participant heard about and accessed the registry survey.

## Registry Workflow

**Fig. 1.**
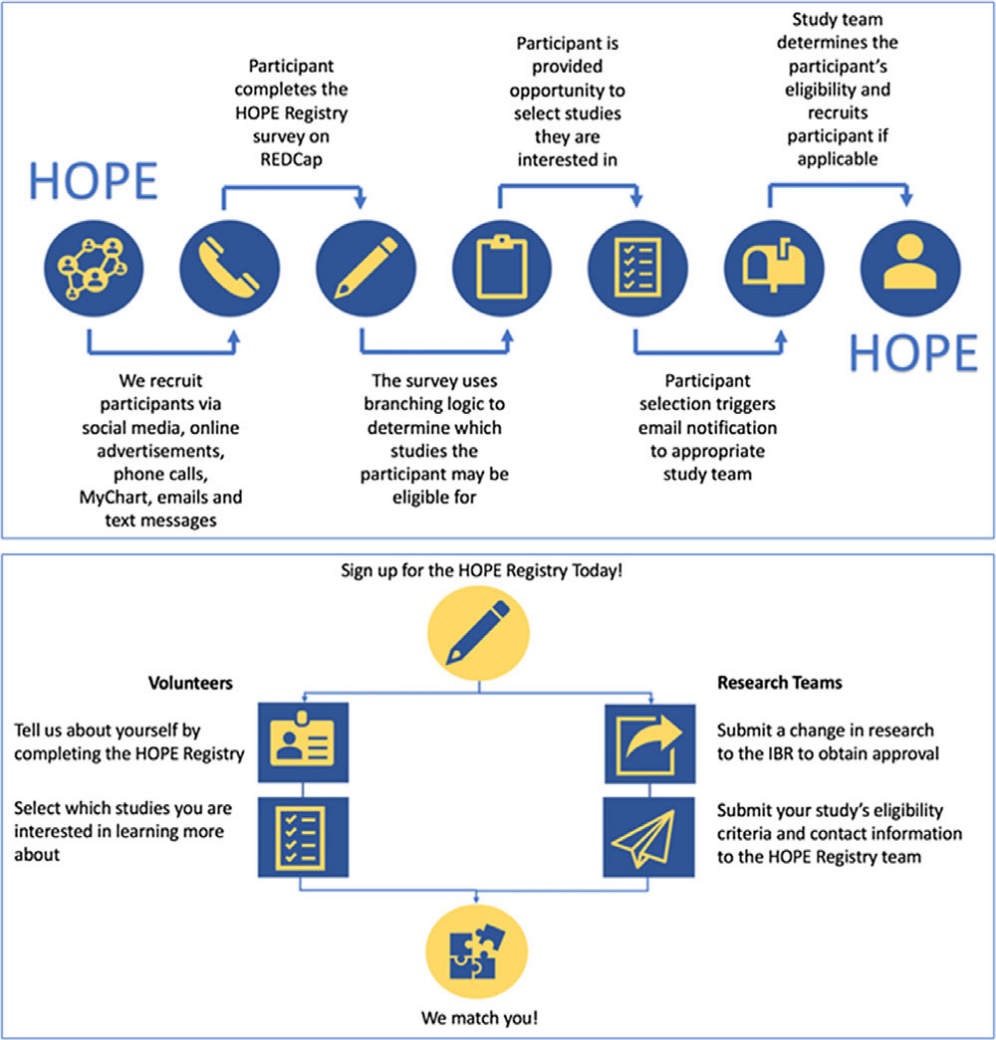
Registry workflow. HOPE, Hopkins Opportunities for Participant Engagement; IRB, institutional review board; REDCap, Research Electronic Data Capture.

The registry workflow is summarized in Fig. 1. In brief, registry participants self-enroll by answering several demographic and COVID-19-related questions. Based on the participants’ responses to these questions, the registry uses branching logic to pre-screen participants for participating studies. At completion of the survey, participants are able to view studies that they are eligible for and if desired, indicate interest in learning more. When they express interest in a study, an email notification is sent to members of the corresponding study team. The study team then reaches out to the registry participant, assesses their eligibility for the study, and completes an outcome survey within REDCap. Additional details on these steps are described below.

### Participant Enrollment and Survey Completion

REDCap, a secure web-based application, serves as the platform for the HOPE Registry.^17,18^ Upon entry to the registry survey, participants have the option to select their preferred language (English or Spanish). The participants can choose to switch their language at any point during survey completion. A language-specific e-consent is included on the first page of the REDCap survey. The e-consent includes a description of the registry and a list of the risks and benefits associated with the registry. Participants are required to provide consent before answering any of the survey questions. Signing the consent within the registry survey does not imply consent for any of the individual studies that recruit from the registry.

Following consent, participants answer a series of questions related to their demographics, exposure to COVID-19, testing, and willingness to participate in different types of research. A list of included questions is shown in the Supplementary Materials. The branching logic feature in REDCap was used to facilitate the matching process. This feature allows the database developer to program a set of conditions that must be met in order for a registry participant to be shown information about a study. First, study teams provided the developer with a list of eligibility criteria for their study. Second, the eligibility criteria were used to identify questions within the registry that could guide the programing branching logic. For example, if a study required participants to be COVID-19 positive, the branching logic would be programmed to only display that study description if a participant selected they were COVID-19 positive on the registry survey. The majority of our study teams specified 3–6 key eligibility criteria to be used in this process.

At completion of the survey, participants are presented with a short description of studies for which they might be eligible. Registry participants then have the option to select which studies, if any, they are interested in learning more about. Expressing interest does not mandate participation in any study. If a registry participant is not eligible for any studies at the time of enrollment, they are sent information about new studies that they might be eligible for as new studies are added to the registry.

### Participant Engagement

The HOPE Registry team aims to keep participants engaged with the registry by soliciting feedback about our processes, informing them about new studies for which they might be eligible, and providing easy access the latest news related to the COVID-19 pandemic. The HOPE Registry Advisory Council prioritizes the experience of community volunteers and research participants and aims to meet the needs of both researchers and the Baltimore community. As such, a satisfaction survey has been distributed to HOPE participants to better understand the participants’ experience with the registry and to engage them in the research process. We will continue to administer the satisfaction survey to new participants quarterly. The results will be used to continue to refine survey questions, develop engagement strategies, and improve the workflow.

To keep registry participants informed, a Microsoft Sway advertisement is created and distributed via email each week. Microsoft Sway is an easy to use digital storytelling application that is part of the Microsoft Office family of products. The Sway advertisement contains information about research related to COVID-19 being conducted at our institution, pertinent news stories relevant to the pandemic and strategies to keep people safe, and resources for how to get vaccinated. Sway is a Microsoft Office app that allows users to create interactive reports and presentations that can be opened, read, and shared by anyone with access to the link.^19^

## Preliminary Results

The HOPE Registry was launched on July 24, 2020. As of March 24, 2021, 9010 participants have enrolled and completed the registry survey. The socio-demographic characteristics of currently enrolled participants are reported in Table 1. The mean age is 53.3 (±16.6), and approximately 18.6% of participants had tested positive to COVID-19 on registry enrollment. Females represent 64.2% of registry participants, and 20.7% are health care workers. The majority of participants are White (80.6%); 9.4%, Black or African American; 4.7%, Asian; 0.4%, American Indian or Native Alaskan; 0.1%, Native Hawaiian or Pacific Islander, and 4.7% self-reported as other. Approximately 5.6% of participants reported their ethnicity as Hispanic or Latino. Since the introduction of the Spanish language version of the HOPE Registry on September 23, 2020, 247 participants (2.3%) have enrolled using the Spanish language version.

**Table 1.** Hopkins Opportunities for Participant Engagement (HOPE) Registry demographics

	Number	Percentage of total
Total enrolled	9010	
Matches	4596	
Mean age in years (SD)	53.3 (±16.6)	
Have tested positive for COVID 19	1541	18.60
Sex		
Female	5775	64.20
Male	3195	35.50
Other or prefer not to answer	19	0.30
Ethnicity		
Not Hispanic or Latino	8260	93.40
Hispanic or Latino	492	5.60
Other	94	1.00
Race		
American Indian or Native Alaskan	35	0.40
Asian	424	4.70
Black or African American	844	9.40
Native Hawaiian or Pacific Islander	12	0.10
White	7240	80.60
Other	428	4.70
Health care worker	1822	20.70

SD, standard deviation.

Enrollment metrics are presented in Table 2. Response rates were calculated by dividing the total number of responses by the total number of contacts for each method. Phone calls from navigators have had the highest response rate with 13% of contacted individuals signing up for the registry, followed by email (11.9%), text (11.4%), and patient portal message (9.4%). The majority of participants enrolled from a link in a patient portal message (70.9%) followed by email (8.7%), text messages (8.3%), social media advertisements (5.5%), Sway advertisements (5.4%), postcards from testing sites (0.9%), and phone calls from recruitment navigators (0.2%). As a note, an individual participant may have received multiple outreach attempts based on the contact information listed in the EMR. The enrollment data reflect the method from which the participant clicked the link and joined the registry.

**Table 2.** Hopkins Opportunities for Participant Engagement (HOPE) Registry enrollment rate by contact method*

Contact method	Number of receiving messages	Enrolled	Enrollment rate (%)
Patient portal	90,839	9180	9.38
Email	8818	1130	11.93
Text	8794	1075	11.37
Call	215	31	13.03

* Individuals may have received messages from more than one contact method.

**Fig. 2.**
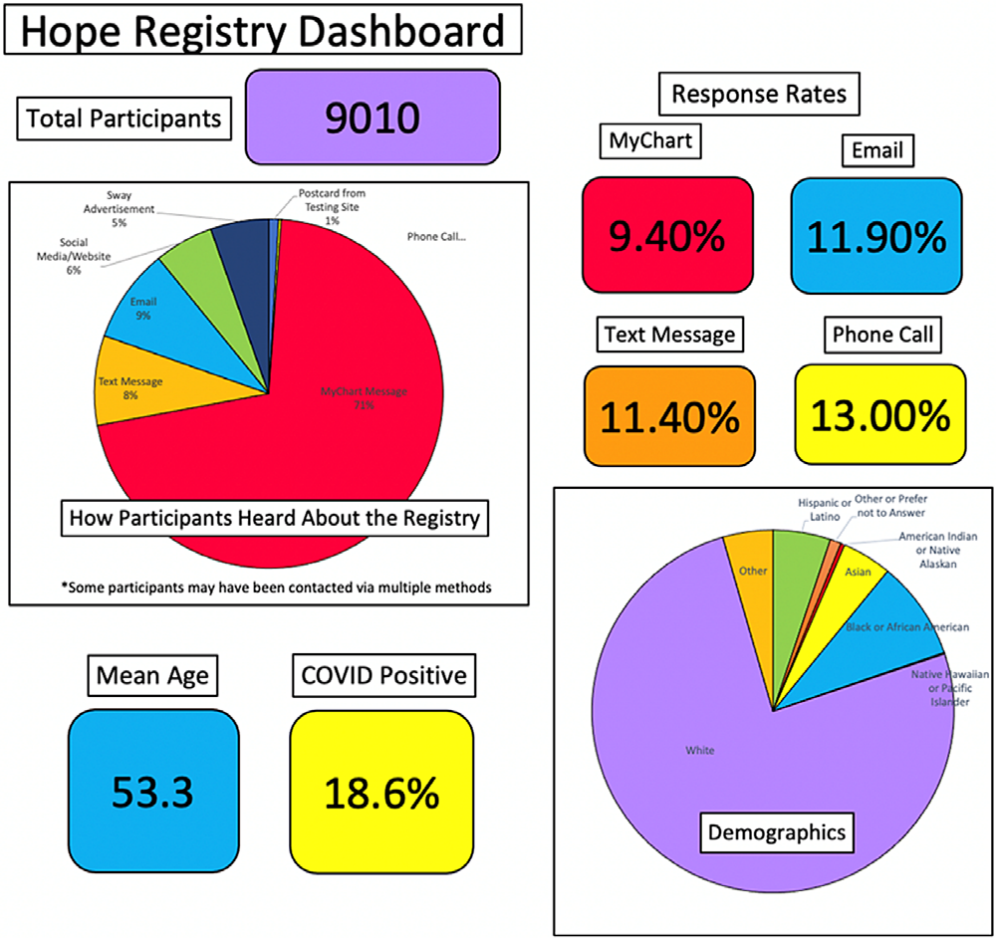
Hopkins Opportunities for Participant Engagement (HOPE) Registry dashboard.

Eleven study teams have utilized the HOPE Registry as a recruitment tool. Nearly 4600 matches have been made between study teams and interested volunteers as of March 24, 2021. Some participants have expressed interest in more than one study. A user-friendly dashboard used to report results of the registry to study teams and administration is shown in Fig. 2.

Currently, the HOPE Registry requires one full-time program manager, two part-time coordinators, and one part-time IT manager. In total, web development, technology support, and recruitment materials have cost around $32,000. This funding was provided by the Johns Hopkins ICTR. The HOPE Registry is available to study teams at our institution at no cost.

## Discussion

The primary goal of the registry was to connect volunteers interested in participating in research related to COVID-19 with actively recruiting outpatient COVID-19 research studies. In our early experience, the HOPE Registry has been effective in identifying people who are interested in participating in research and connecting them with research teams seeking participants. Since inception, over 9000 individuals interested in participating in COVID-19-related research have joined the registry, and nearly 4600 matches have been made between study teams and interested volunteers. This has aided 11 outpatient studies in their recruitment. Because 9 of the 11 studies who have used the HOPE Registry for recruitment are still actively recruiting, the number of people enrolled in studies related to COVID-19 as a results of the HOPE Registry is incomplete. We will present final enrollment data in future reports.

Importantly, our approach increased awareness and access within the community regarding the availability of COVID prevention and therapeutic trials. As a digital recruitment tool, HOPE Registry has facilitated recruitment and improved efficiency for 11 study teams by centralizing multiple processes that would otherwise be redundant across teams. The HOPE Registry also reduced the burden on potential participants, most of whom had recently been tested for COVID, by coordinating the recruitment efforts of multiple studies.

While the HOPE Registry has been successful in recruiting thousands of people interested in COVID-19-related research, there have been challenges engaging a diverse sample. Baltimore City is 64% Black or African American, yet only 9% of the registry enrollees are Black or African American.^20^ After decades of exploitation of the Black community in medical research in the USA, particularly at Johns Hopkins, there is still a mistrust of researchers and research institutions.^21,22^ This could have contributed to the lack of representation of Black and African American participants. Moreover, the focus on digital recruitment strategies could have favored White, educated, middle, and upper class participants and contributed to the bias of the sample.^23^ Additionally, with around 20% of the HOPE Registry participants being health care workers, Johns Hopkins employees are over represented. We have sought input from our partners in the C-RAC to increase the representation of Black and African American registry participants. Their recommendations have included creating open forums for community members to ask questions about COVID-19 and the research process, making the language of the registry and the included studies easier to understand, engaging faith-based communities to spread the word, and continuing to engage with those interested in research and those who have participated to share results whenever they are available.

COVID-19 has hit Hispanic communities in the USA especially hard. Hispanic adults are three times more likely to contract COVID 19 and nearly five times more likely to be hospitalized as a result.^3^ This has created new and worsened existing health disparities. Though Hispanics are the largest minority group in the USA with about 18% of the population, they are grossly underrepresented in research.^24,25^ In an effort to reach the Hispanic community to increase representation in research related to COVID-19, the HOPE Registry team collaborated with Centro SOL, a division of Johns Hopkins Medicine whose mission is to promote equity in health and opportunity for Latinos by advancing clinical care, research, education, and advocacy. Centro SOL provided translations to create the Spanish language survey and outreach messages. Additionally, they provided key guidance to help engage the Latino community with the registry’s mission and educated about research related to COVID-19. Thanks to these efforts, around 6% of registry participants identify as Hispanic or Latino, which is representative of Baltimore city’s demographics.^26^ However, we recognize that this does not represent the population impacted by COVID-19. Positivity rates reached more than 42% in the Latino population in our health system.^27^ Therefore, we are working closely with Centro SOL on cultural and language appropriate adaptations and refinement of the recruitment of Spanish-speaking participants in the HOPE Registry.

Due to COVID-19 restrictions, to date, nearly all recruitment has been conducted remotely to prevent unnecessary contact between research team members and potential participants. The HOPE Advisory Council responded with a robust digital recruitment platform, allowing the registry to reach a large number of community members without in-person contact. However, the use of primarily digital recruitment limits the ability to reach individuals without access to computers or the internet. The HOPE Advisory Council has attempted to combat this by exploring outreach methods that do not require internet access, such as postcards at testing sites and recruitment navigator outreach over the phone. Despite these efforts, the limited ability to reach people without internet access is a limitation of the registry. We anticipate that as COVID restrictions are relaxed, we will be able to complement digital with in-person strategies to support engagement with the HOPE Registry.

### Implications and Future Directions

These preliminary findings have meaningful implications for future clinical research and research recruitment. First, we demonstrated the ability to implement a multi-study recruitment campaign through the use of primarily digital methods. These approaches are increasingly important to leverage during the COVID-19 pandemic, where in-person recruitment poses a safety risk to the research staff and research participants. Second, our approach successfully connected interested and eligible participants with actively recruiting study teams, eliminating the potential burden participants might face by receiving recruitment emails or phone calls from several study teams. Further, it was centered around providing the participant with autotomy to choose studies that fit their needs, interests, and comfort levels, rather than allowing health care providers or research personnel to choose on behalf of the potential participant. Given the unprecedented number of studies focusing on the same disease state and patient population, this approach was especially important to ensure participants were aware of their options and were able to make an informed decision on their participation.

While the HOPE Registry was initially created out of necessity during the early days of the COVID-19 pandemic, we hope to transition the HOPE Registry to a wider research registry recruiting and matching healthy volunteers and volunteers with all types of conditions to Johns Hopkins researchers. We plan to follow a structure similar to Pitt + Me research registry and are actively working with their team to create the infrastructure necessary to expand our current base.^16^ Plans have begun, and we hope to launch the wider registry by fall 2021. We will obtain IRB approval and reconsent all participants who would like to be part of this larger registry.

## Conclusion

Effective and efficient research recruitment strategies are critical to ensure the timely and successful completion of COVID-19 research. Preliminary results of the HOPE Registry have indicated that this approach has significant promise to providing opportunities for individuals to engage with COVID-19 research and improve research recruitment workflow.

## Supplementary material

For supplementary material accompanying this paper visit http://dx.doi.org/10.1017/cts.2021.819. click here to view supplementary material
