# Supplementary material for: Centralized registry for COVID-19 research recruitment: Design, development, implementation, and preliminary results
Source: J Clin Transl Sci. 2021 Jul 14;5(1):e152. doi: 10.1017/cts.2021.819 (PMC8387691; doi:10.1017/cts.2021.819)
Supplement: Supplementary file 1 [file S2059866121008190sup001.docx]

1. Survey Completion Date

2. Preferred Language

- English
- Español

3. Contact Method

- Postcard from testing site
- Outreach from RIU Navigator
- MyChart message
- Text message code
- Email
- Social Media/Website
- Other advertisements
- Text message outreach
- Sway advertisements
- Spanish Outreach

Introduction:

**What is the COVID-19 Research Registry and why are we using it?**

The COVID-19 Research Registry is a tool being used at Johns Hopkins to connect eligible and willing volunteers with COVID-19 related research studies at Johns Hopkins. Because we have much to learn about the prevention, diagnosis and treatment of COVID-19, there are a number of COVID-19 related research studies at Johns Hopkins.

**How do I volunteer to be a part of the COVID-19 Research Registry and how does the Registry work?**

To volunteer to be in the COVID-19 Research Registry, you must complete a short survey. This survey will take approximately 5 minutes and will ask you questions about your demographics, exposure to COVID-19 and willingness to participate in different types of research. Your responses will be used to match you to an appropriate study. These studies will be reviewed by a Johns Hopkins Institutional Review Board (IRB). If you match study criteria, a research team member will reach out to you via your preferred method of contact. Completing this survey does NOT mean that you have to participate in a study if you are contacted. You may be matched to or contacted by more than one study.

Your responses will be stored securely. You do not have to complete all components of the survey if you wish not to. Only information completed in the survey will be utilized to match you to existing research studies.

**What are the benefits and risks to participating in the COVID-19 Research Registry?**

By participating in the COVID-19 Research Registry, you will have the opportunity to partake in research related to the prevention, diagnosis or treatment of COVID-19. This Registry will increase your chances to being matched to research studies that you are eligible for.

We will do our best to protect and maintain your information in a safe way. The risk related to joining this registry may be a loss of confidentiality of the information you provide. We have taken steps to reduce the risk. All information you provide will be stored and shared only using secure methods. All staff seeing your information have required institutional privacy training and research study teams receiving information have the same training.

There may be risks to participating in studies. If you are contacted about joining a study, the researchers will review the risks and benefits of the study with you in detail before you decide if you would like to join.

By participating in this survey, you are giving Johns Hopkins permission to collect your contact information. This information will be used to determine if you may be eligible to join COVID-19 research being conducted by Johns Hopkins.

Your privacy is important to us. Any data provided will be maintained on secure, HIPAA (Health Insurance Portability and Accountability Act) compliant systems. The information will be used to determine if you are a possible match for COVID-19 research by Johns Hopkins.

Participation in this registry is voluntary. You will be able to participate in or decline any study offered to you. If you do not wish to provide your private information, you may leave the survey at this time.

NOTE: Only residents of the United States may complete the survey

- Yes, I acknowledge the above and wish to provide my personal and health information as a survey participant
- No, I do not want to participate in this survey

4. First Name

5. Last Name

6. Date of Birth

7. What sex were you assigned at birth?

- Female
- Male
- Unknown
- Other
- Prefer not to answer

8. Are you of Hispanic, Latino, or Spanish origin?

- No, not of Hispanic, Latino, or Spanish origin
- Yes, of Hispanic, Latino, or Spanish origin
- Unknown
- Prefer not to answer

9. How would you identify your Race?

- American Indian or Alaska Native
- Asian
- Black or African American
- Native Hawaiian or Pacific Islander
- White
- Pacific Islander
- Latino or Latina
- Other
- Unknown
- Prefer not to answer

10. Street Address

11. City

12. Zip Code

13. I prefer to be contacted by (check all that apply):

- Email
- Phone
- Text

14. Email Address

15. Phone Number

16. How did you find out about this research interest tool?

- Referral from health care worker
- Referral from someone else
- Saw a brochure or flyer
- Web search
- Facebook ad
- Community group
- MyChart
- Other

17. Have you been exposed to someone with COVID-19, such as a person or persons with whom you live or work with?

- Yes
- No

18. Was this exposure greater than or equal to 24 hours?

- Yes
- No

19. What was your approximate first date of exposure?

20. Are you a healthcare worker?

- Yes
- No

21. If yes, are you exposed to patients with COVID-19?

- Yes
- No

22. Have you had symptoms of COVID-19 (cough, fever, body aches, or loss of taste or sense of smell) since March 1, 2020?

- Yes
- No

23. If yes, what was the approximate date that you first noticed these symptoms?

24. Have you ever taken any treatment to prevent or treat COVID-19?

- Yes
- No

25. If yes, what did you take?

26. Have you been tested for COVID-19?

- Yes
- No

27. If yes, what was the approximate date of your COVID-19 laboratory testing?

28. What were the results of your COVID-19 laboratory testing?

- Negative
- Positive
- I do not know my results

29. Why were you tested?

- I had symptoms of COVID-19 illness
- I was exposed to someone who had COVID-19 illness
- I was tested for another reason
- I was in the hospital for COVID-19

30. Have you been hospitalized for COVID-19?

- Yes
- No

31. How many children under 18 years of age live in your home?

32. Would you be interested in a study that involves taking a medication to prevent or treat COVID-19?

- Yes
- No
- Maybe, I need more information

33. Would you be interested in a study of a vaccine to prevent COVID-19?

- Yes
- No
- Maybe, I need more information

34. If you have had COVID-19, are you interested in donating your plasma for use in research studies?

- Yes
- No
- Maybe

35. Would you be willing to (check all that apply):

- Travel to a research center in your area?
- Volunteer for multiple research visits spaced out over time?
- Volunteer for a single research visit?
- Participate in a study done remotely over the phone, over the Internet, or over the computer?

36. How many people live with you?

37. How many people over the age of 60 years live in your home?

38. Do you think that people in your household should be tested for COVID-19?

- Yes
- No
- Maybe

39. Is there any other information you would like to share with us?

We sincerely thank you for your interest in supporting research efforts to fight COVID-19. Based on your responses, you may be interested in the following list of studies. If you select you are interested, a study team member from the project will reach out to you via your preferred method of contact to tell you more about the study. You do not have to participate in the study.

Study 1: This study is testing the use of an investigational drug Lambda Interferon (not FDA approved for any purpose) in the prevention of SARS-CoV-2 infection in people who have been exposed through household contact. Lambda is an injection into the skin that is given two times, one week apart. The length of study participation is 28 days, with a phone or remote contact on day 84. Participants will receive $50 for completing all visits.

I am interested in learning more about this study (check if you are interested)

Study 2: This study will test whether a transfusion of plasma containing antibodies from persons who have recovered from COVID-19 can prevent others from getting COVID-19 after exposure. This is a randomized, double-blind trial. Participants will receive plasma containing SARS-CoV-2 antibodies or plasma without antibodies.

I am interested in learning more about this study (check if you are interested)

Study 3: This study is recruiting persons who have tested POSITIVE for COVID-19 and willing to donate their plasma (a part of your blood) for research. Their plasma would be used to treat COVID-19 in patients who have it now or help others at risk from getting COVID-19. The study would also conduct a test of virus in participant's blood to learn more about COVID-19.

I am interested in learning more about this study (check if you are interested)
